# Combined Role of Seizure-Induced Dendritic Morphology Alterations and Spine Loss in Newborn Granule Cells with Mossy Fiber Sprouting on the Hyperexcitability of a Computer Model of the Dentate Gyrus

**DOI:** 10.1371/journal.pcbi.1003601

**Published:** 2014-05-08

**Authors:** Julian Tejada, Norberto Garcia-Cairasco, Antonio C. Roque

**Affiliations:** 1 Departamento de Física, Faculdade de Filosofia, Ciências e Letras de Ribeirão Preto, Universidade de São Paulo, Ribeirão Preto, São Paulo, Brasil; 2 Departamento de Fisiologia, Faculdade de Medicina de Ribeirão Preto, Ribeirão Preto, São Paulo, Brasil; Université Paris Descartes, Centre National de la Recherche Scientifique, France

## Abstract

Temporal lobe epilepsy strongly affects hippocampal dentate gyrus granule cells morphology. These cells exhibit seizure-induced anatomical alterations including mossy fiber sprouting, changes in the apical and basal dendritic tree and suffer substantial dendritic spine loss. The effect of some of these changes on the hyperexcitability of the dentate gyrus has been widely studied. For example, mossy fiber sprouting increases the excitability of the circuit while dendritic spine loss may have the opposite effect. However, the effect of the interplay of these different morphological alterations on the hyperexcitability of the dentate gyrus is still unknown. Here we adapted an existing computational model of the dentate gyrus by replacing the reduced granule cell models with morphologically detailed models coming from three-dimensional reconstructions of mature cells. The model simulates a network with 10% of the mossy fiber sprouting observed in the pilocarpine (PILO) model of epilepsy. Different fractions of the mature granule cell models were replaced by morphologically reconstructed models of newborn dentate granule cells from animals with PILO-induced *Status Epilepticus*, which have apical dendritic alterations and spine loss, and control animals, which do not have these alterations. This complex arrangement of cells and processes allowed us to study the combined effect of mossy fiber sprouting, altered apical dendritic tree and dendritic spine loss in newborn granule cells on the excitability of the dentate gyrus model. Our simulations suggest that alterations in the apical dendritic tree and dendritic spine loss in newborn granule cells have opposing effects on the excitability of the dentate gyrus after *Status Epilepticus*. Apical dendritic alterations potentiate the increase of excitability provoked by mossy fiber sprouting while spine loss curtails this increase.

## Author Summary

Neurogenesis is currently a well known phenomenon in the adult brain, in special in some areas such as the subventricular zone and the dentate gyrus in the hippocampus, in which different endogenous and exogenous factors provoke cell proliferation. In the specific case of the dentate gyrus, granule cells proliferate exhibiting altered morphology after the induction of *Status Epilepticus* (SE) by pilocarpine (PILO). Several days after the injury the new cells show different morphological alterations, for example, in dendritic spines and branching patterns, as well as with the formation of axonal sprouting. The way in which these new cells are integrated into the hippocampus is still unknown with conflicting data in the literature. Here we used computer simulation to test if the activity of the dentate gyrus is affected by the presence of different proportions of new cells after PILO-induced SE. Our results show that the specific morphological alterations present in the granule cells in rats with PILO-induced SE may be responsible for increasing (mossy fiber sprouting) or decreasing (spine loss) the activity in the network. The imbalance between these effects may be manifest as an epileptiform network behavior.

## Introduction

Several evidences have shown that prolonged seizures such as those in the animal models of *Status Epilepticus* (SE) induced by kainic acid or pilocarpine [1] act as a strong insult with consequent anatomical and functional sequelae. The neuroplastic changes associated to these alterations include cell loss, dentate gyrus (DG) granule cells (GCs) dispersion, mossy fiber sprouting (MFS), dendritic spine loss, neurogenesis with dendritic branching pattern alterations and the presence of newly generated cells in ectopic places [2]–[8]. Although there is still some controversy on how much each one of these alterations contributes to epileptogenesis, it is clear that multivariate interactions are needed for the full appearance of the chronic state with spontaneous recurrent seizures (SRS) [9]. Basically these models replicate cellular and molecular plastic alterations, besides epileptiform EEG features observed in clinical TLE [10]–[12].

In this scenario, some of the most studied phenomena are the plastic alteration of the circuits of the DG GCs. In fact, DG GCs present a series of morphological and anatomical alterations induced by temporal lobe epilepsy (TLE) [6], [7], [13]–[17]. Their axons sprout collaterals directed towards the DG molecular layer and make recurrent excitatory synapses with other GCs (this phenomenon is called MFS) [2], [6]. The size of their soma and the shape of their apical dendritic trees are altered, exhibiting shorter and narrower dendrites [16]. Furthermore, those newly born neurons display a significant reduction in the number of dendritic spines [7], [18], [19]. In some cases, there is a basal dendrite that sprouts towards the DG hilus [7], [17]. There are also GCs that migrate to ectopic places such as the hilus and granular cells layer [6], [13]. In parallel with these morphological alterations, TLE also induces a higher rate of neurogenesis of DG GCs [20], [21].

Mossy fiber sprouting is the most widely studied of the above mentioned alterations using different approaches including animal models [22], [23], cell cultures [24], [25], knockout genes [26], [27] and computational models [28]–[30]. These studies showed that mossy fiber sprouting is an important factor contributing to the hyperexcitability of the hippocampal circuit [22], [23], [25], [28], [31], [32]. On the other hand, the effects of the other alterations on the excitability of the DG remain unclear, in special the effects of alterations in dendritic morphology and the combined effects of the interacting factors.

Neuronal morphology is an important factor in the determination of the electrophysiological behavior of a cell [33], [34]. However, due to its inherent complexity, it is difficult to assess experimentally the effect of neuronal morphological alterations on the behavior of a cell or of the circuit in which it is embedded. Computational modeling enters here as a valuable tool to assess this effect and many studies have been concerned with the evaluation of the coupling between neuronal morphology and electrophysiological behavior [34]–[38].

In a previous research [39], [40] we studied the effect of morphological alterations in the apical dendritic trees of isolated GCs on their excitability using computational models built from three-dimensional reconstructions of GCs of animals that had *Status Epilepticus* (SE) induced by pilocarpine (PILO) and control animals. The models in the two groups (PILO and control) have the same distributions of ionic channels and the same maximal conductance densities over their dendritic areas measured according to their distance from soma (proximal, medial and distal). With this approach we aimed to evaluate the effect of morphological alterations alone on single-cell excitability. We found that GCs with altered morphology are less excitable when stimulated in a simulation of a patch clamp protocol [39].

In the present work, we extended our single-cell study to a network context (a collection of neurons) to obtain information on the effect on DG hyperexcitability of seizure-induced apical dendritic morphological alterations in GCs in addition to dendritic spine loss. We use a previously described network DG model, which incorporates detailed structural and biophysical information on DG network and cells [28]. In their work, Santhakumar et al. [28] studied the effect of mossy fiber sprouting on the spread of activity through the DG network after simulated focal stimulation of the perforant path. They showed that even weak mossy fiber sprouting, e.g. 10% of the one observed in the PILO model of TLE, caused the activity to spread from the directly stimulated GCs to the entire network in a seizure-like fashion.

The model of Santhakumar et al. [28] uses reduced compartmental models of DG granule, mossy, basket and hilar cells, as well as entorhinal perforant path-associated stimulation signals. Their GC model is highly simplistic and has only two dendrites, whereas ours replaces these GC models with morphologically realistic compartmental models based on data from three-dimensional cell reconstructions (downloaded from neuromorpho.org). To do so we had to recreate the connections between GCs and the other cell types (which were kept as in the original model). Our model has the same network structure of the model of Santhakumar et al. [28] with 10% mossy fiber sprouting. We used morphologically reconstructed models of mature and newborn GCs. The latter came from control animals that did not receive drug injections and from PILO-treated animals that exhibited SE. The models from animals with SE have morphological alterations in their apical dendrites in comparison to models of the control group [16]. We submitted networks with different percentages of newborn cells, either exclusively from the control group or exclusively from the PILO-treated group, to the same focal stimulation protocol used by Santhakumar et al. [28].

Thus, the objective of our study was to obtain mechanistic insights on the integrated action of three types of GC seizure-induced morphological alterations, namely mossy fiber sprouting, apical dendritic tree alterations and dendritic spine loss, on DG hyperexcitability.

## Methods

We used a 1∶2000 scaled-down model of the dentate gyrus [28] with the reduced GC models replaced with morphologically realistic models to evaluate the impact of morphological variations on the excitability behavior of the DG.

**Figure 1:**
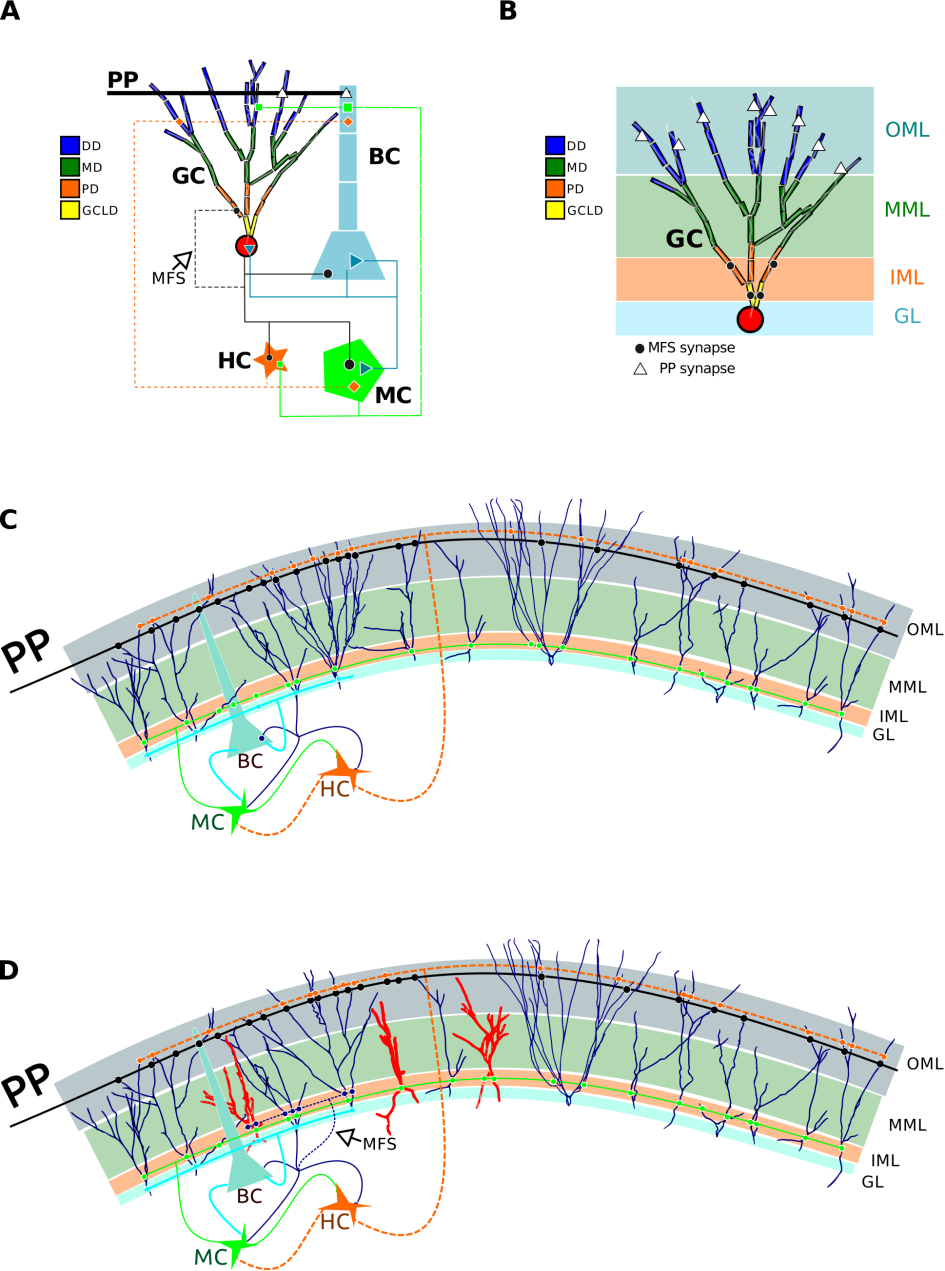
Scheme of the structure and topology of the network. **A**. Schematic representation of network connections between granule cells (GC), basket cells (BC), hilar perforant-path associated cells (HC) and mossy cells (MC). GC synapses are indicated by black lines, BC synapses are indicated by blue lines, HC synapses are indicated by orange dashed lines and MC synapses are indicated by green lines. Mossy fiber sprouting (MFS) is indicated by a black dashed line and the perforant-path input to GC and BC is indicated by a bold black line. The segments into which the GC dendritic tree are divided are indicated in yellow (granule cell layer dendrites), orange (proximal dendrites), green (medial dendrites) and blue (distal dendrites). **B**. Positioning of the GC within the molecular layer with its four subdivisions: granular layer (GL) in blue, inner molecular layer (IML) in orange, middle molecular layer (MML) in green and outer molecular layer (OML) in blue. The figure shows that perforant-path synapses to GCs are located in OML dendrites and mossy fiber sprouting synapses to GCs are located in IML dendrites regardless of the type of dendritic segment in these layers. **C**. Schematic representation of the dentate gyrus model with control GCs. **D**. Schematic representation of the dentate gyrus model with a fraction of the control GCs replaced with cells from PILO-treated animals (in red).

The network model contains 500 granule cells (GC), 6 basket cells (BC), 15 mossy cells (MC) and 6 hilar perforant-path associated cells (HC), arranged in a ring structure with topographic connections between cells (Figure 1). Each MC contacts 200 GCs, 1 BC, 2 HCs and 3 other MCs. Each HC connects with 160 GCs, 4 BCs and 4 MCs. Each BC contacts 100 GCs, 3 MCs and 2 other BCs. In normal conditions each GC contacts 1 MC, 1 BC and 2 HCs, but when there is MFS, the GCs make contacts with other GCs. The MFS is quantified by the number of connections from each GC to other GCs (Figure 1C). This DG model has been used to evaluate the propagation, across the 500 GCs, of a stimulation pattern from the entorhinal cortex which reaches only the first 100 GCs (Figure 1).

Our model of DG was identical to the model by Santhakumar et al. [28] available on ModelDB (http://senselab.med.yale.edu/ModelDb/ShowModel.asp?model=51781) for the condition with 10% of MFS. The only change we made was to replace the two dendrites model of GC by another with realistic morphology from a sample of 74 normal GCs three-dimensional reconstructions available in neuromorpho.org. The sample consists of neurons reconstructed by the groups of Brenda J. Claiborne [41], Dennis A. Turner [42], Joseph P. Pierce [43], Vijayalakshmi Santhakumar [44] and Giorgio Ascoli [45]. All of them were chosen because they come from studies of morphological characterization in which drugs were not used to alter the cell morphology, and also because these cells present the typical cone shape and size observed in adult GCs. From the total of 93 available rat GC reconstructions, we only used 74 (see Table 1 with the name of the used models), discarding some of the biggest and smallest models and the ones that in a visual inspection did not look like a typical granular cell, trying to get a homogeneous set of models that can be used as a comparison group to the cell with altered morphology.

**Table 1:** List of the three-dimensional GC reconstructions from neuromorpho.org used to build the mature DG model0.

Model name	Lab	Model name	Lab
gc	Ascoli	B106885	Claiborne
124893b	Claiborne	B330886	Claiborne
124894	Claiborne	103-5	Pierce
404881	Claiborne	124-5R	Pierce
410884	Claiborne	5-20-2011cell1-GCcontrol	Santhakumar
410885	Claiborne	n220	Turner
411883	Claiborne	n221	Turner
411884a	Claiborne	n222	Turner
411884b	Claiborne	n223	Turner
418882	Claiborne	n224	Turner
418883	Claiborne	n226	Turner
428883	Claiborne	n241	Turner
512882	Claiborne	n242	Turner
523886	Claiborne	n244	Turner
524892a	Claiborne	n270	Turner
524892b	Claiborne	n272	Turner
601886	Claiborne	n500	Turner
609885	Claiborne	n501	Turner
614882	Claiborne	n502	Turner
720884	Claiborne	n503	Turner
725883a	Claiborne	n504	Turner
725883b	Claiborne	n505	Turner
728882	Claiborne	n506	Turner
803884	Claiborne	n507	Turner
803887b	Claiborne	n508	Turner
805881	Claiborne	n509	Turner
815885	Claiborne	n510	Turner
817884	Claiborne	n511	Turner
817886	Claiborne	n512	Turner
1208875	Claiborne	n513	Turner
1220882a	Claiborne	n514	Turner
1220882b	Claiborne	n515	Turner
1220883	Claiborne	n516	Turner
3319201	Claiborne	n517	Turner
3319202	Claiborne	n518	Turner
4299202	Claiborne	n244	Turner
5199202	Claiborne	n271	Turner

Regarding the newborn GC models, we used the 40 available reconstructions in *neuromorpho.org* from Arisi & Garcia-Cairasco [16].

**Figure 2:**
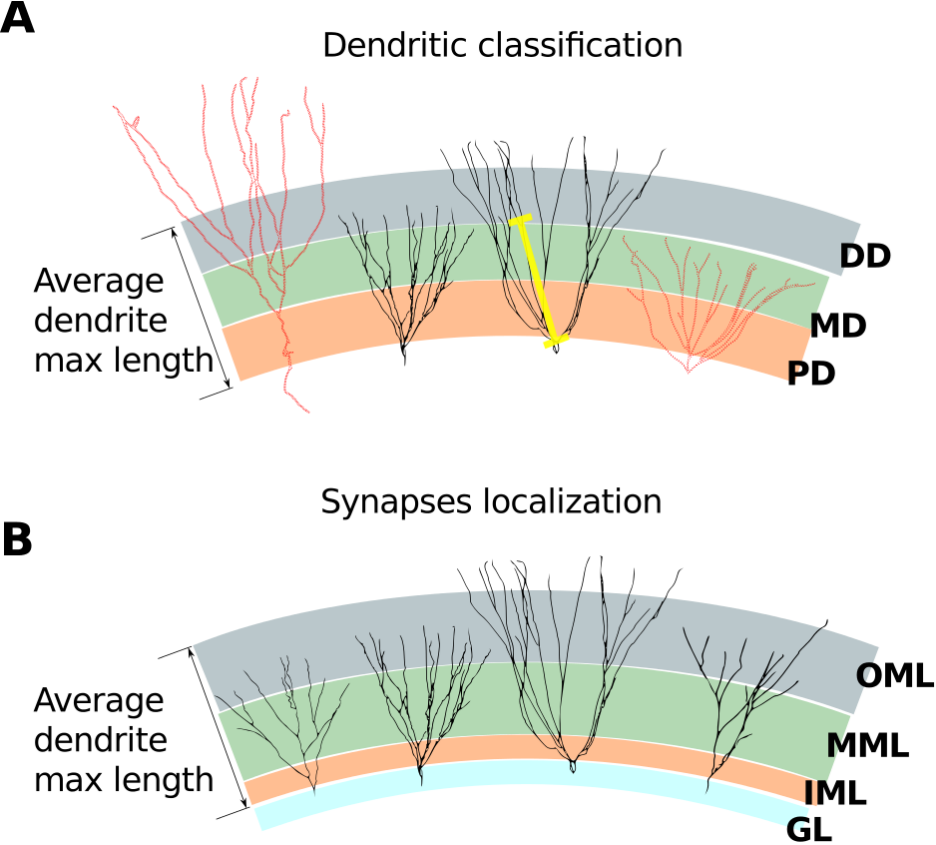
Schematic representation of the strategies used to classify the dendrites and place the synapses. **A**. Classification of a dendrite as proximal (PD), medial (MD) and distal (DP) based on the distance from soma up to the branching point of the dendrite (yellow line). **B**. Location of the synapses depending on the sublayer in which a dendrite section is located.

The GC computational models were constructed following the methodology described in Tejada et al. [39], in which we consider the same ion channel conductances, densities and distribution and synapses in all of the models of the GCs, using the pruned-distance 1 criterion, described by Tejada et al. [39], for the classification of the dendritic tree. This criterion divided the dendritic tree into four segments: granule cell layer dendrites, (GCLD) proximal dendrite (PD), medial dendrite (MD) and distal dendrite (DD) (Figures 1B and 2), and classified the dendrites according to the average maximum values of the distance measure from soma to the branching point of each dendrite. None of the morphological reconstructions have axons, with exception of the models from Ascoli's group [45]. For this reason axons were not explicitly simulated and were replaced by conductions delays following the original model of Santhakumar et al. [28]

For the location of the synapses it was considered a virtual molecular layer with thickness equal to the average maximum dendritic length observed in the three-dimensional models plus and minus the standard error (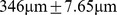). This virtual molecular layer was divided into three regions following Murphy et al. [7]: the first 17% nearest to the granular cell layer (Figure 2B) was denominated inner molecular layer, the remaining 83% was subdivided into two equal size layers: middle and outer molecular layers. The synapses were located at the middle points of the sections of the apical dendrites within each of the three molecular layers. It is important to note that the criterion to classify the dendritic tree into proximal, medial and distal do not necessarily matches the criterion used to locate the synapses (Figure 2) and, in some cases, it depends on the dendritic length. The same dendrite may extended from the inner up to the outer molecular layer, so consequently this dendrite may present different synapses along its length. In this sense, for instance, the synapses that receive the signal from the perforant path and which are generally located in the outer molecular layer were located in each one of the segments of the dendrites that reached this layer, independently of these dendrites being classified as distal or not. The same was made for the synapses located in the inner molecular layer (Figure 2). Some of the three-dimensional GC reconstructions used by us have basal dendrites and we kept them in our models. However, since our network model does not include the hilus we did not consider synapses located on the basal dendrites in the present study.

Despite the increase in the number of synapses due to the increase in the number of dendrites, the connection between cells was maintained similar to the original model of Santhakumar et al. [28] with only one connection between the different kind of cells with the exception of mossy cells that may make more than one connection with the same GC, and also in the case of MFS in which a GC may make more than one connection with some neighboring GC. Following the original model of Santhakumar et al. [28], the connections were randomly raffled choosing at random pre- and post-synaptic cells and also the specific dendrite which receives the presynaptic signal.

Once the model with the three-dimensional cells was established (we called it the mature model), we generated two big families of models with different proportions of seizure-induced neurogenesis (from 0 up to 100%) in which the mature GC models were replaced by three-dimensional reconstructions of newborn (30 days old) GCs. In each of these models the mature GCs that were replaced by newborn cells were chosen at random from the entire population of mature GCs in the network. The sample of newborn cells consists of 40 reconstructions of doublecortin-positive DG GCs (for details see [16]), 20 of them from rats that underwent SE after treatment with PILO and 20 from control rats, all of them reconstructed at the Neurophysiology and Experimental Neuroethology Laboratory (LNNE) of the Physiology Department at the University of São Paulo at Ribeirão Preto, Brazil. The computational models of these cells were also constructed following Tejada et al. [39], [40] using the same pruned-distance 1 criterion, but in this case we used the average maximum values of the distance measure from soma to the branching point of each dendrite of the GC from control animals, by considering that a newborn cell has the same kinds of ion channels located in the same proportional places in which they are found in mature cells. We also used the same average maximum values found for the GCs from control animals in the dendritic tree classification of the GCs from PILO animals, because the use of this value would mean that the dendrites of the cells which were born after the seizure had their ends pruned (see details about this nomenclature in [39], [40]).

On the other hand, the synapses placement followed a similar criterion to the used for the mature models but in this case we used the value of the thickness of the molecular layer measured for each one of the newborn models (values ranged from 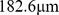 up to 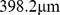) to determine the thickness of the IML, MML and OML, and located the synapses in the middle of the section of each one of the dendrites that extends along these layers.

We also included spine loss in the family of mature networks with newborn GCs from PILO animals. None of the GC reconstructions used in our study (both of mature and newborn cells) have spines implemented explicitly, so we had to represent spine loss in PILO GCs in an indirect way. We chose two ways of doing so here. The default way, present in all spine loss simulations, was done to represent spine loss by a reduction in the probability of connections of the PILO GCs inserted in the model to account for the reduction in dendritic synaptic sites. Three values of probability reduction were considered: 25%, 50% and 75%. The second way, considered together with the reduction in connection probability in some of our simulations, was done to introduce corrections in the membrane resistivity and capacitance of the PILO GC models to account for the reduction in membrane area [46], [47]. These corrections consisted in making the values of dendritic membrane resistivity and capacitance of the cells equal to their somatic values (these values are given in Table 3 of [39]).

To implement the change in the probability of connections of the newborn PILO GCs with spine loss, when the network was built a newborn PILO GC had a reduction of 25%, 50% or 75% of receiving a connection from other cell types in the network. However, a change in the probability of connections can affect the convergence and divergence parameters of the network. Because of this, we simulated two possible scenarios in which spine loss is associated (co-exists) with MFS and dendritic alterations. In the first scenario, which we called spine loss 1 (SL1), we maintained the divergence and convergence parameters of the network by compensating the loss of connections by connecting the cells that would be connected with the newborn GCs that lost spines to other mature cells. In the other scenario (SL2), we did not create new connections with mature cells to compensate for the smaller amount of connections with newborn GCs with spine loss.

Table 2 summarizes the different families of the DG model that were used in the present study. Finally, it is important to mention that for every DG family model the connection pattern among network cells was created anew before any new simulation, and each condition was simulated 20 times for the calculation of averages. The network activity was measured with raster plots and histograms of spike frequency, grouped for differentiating control versus PILO GCs activity and making comparisons using a two-way ANOVA with alpha of 0.01 in all cases. We used NEURON ([48], http://www.neuron.yale.edu) to ran the simulations, with a time-step of 0.1 ms and custom-made Matlab (The Mathworks, Inc., Natick, MA) scripts for data analysis and graphs.

**Table 2:** List of the different families of models developed in the present study.

Family name	Description
Mature	Network models formed by mature GCs from the sample of cells listed in Table 1.
Mature + Young (MY)	Mature network model in which different proportions of newborn GCs from control animals were inserted.
Mature + PILO (MP)	Mature network model in which different proportions of newborn GCs from PILO-treated animals were inserted.
Mature + PILO + SL1 (MPSL1)	Mature network model with different proportions of inserted newborn GCs from PILO-treated animals with spine loss, built maintaining divergence and convergence factors of the mature cells in the network.
Mature + PILO + SL2 (MPSL2)	Mature network model with different proportions of inserted newborn GCs from PILO-treated animals with spine loss, built without maintaining the divergence and convergence factors of the mature cells in the network.
Mature + PILO + SL1 + corrections in membrane properties (MPSL1c)	Same as MPSL1 with spine loss also represented by changes in dendritic membrane resistivity and capacitance.
Mature + PILO + SL2 + corrections in membrane properties (MPSL2c)	Same as MPSL2 with spine loss also represented by changes in dendritic membrane resistivity and capacitance.

## Results

**Figure 3:**
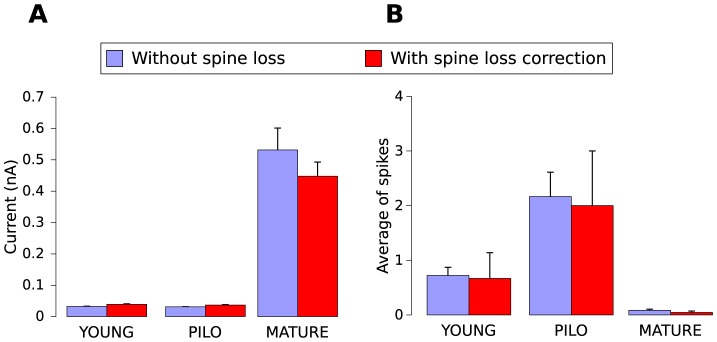
Comparison of the intrinsic excitability of the mature and newborn GCs from control (YOUNG) and PILO groups. The cases for model cells without spine loss are indicated in blue and the cases for model cells with spine loss are indicated in red. **A**. Average rheobase current (measured as explained in the text) for the three model cell groups. **B**. Average number of spikes evoked by the train of synaptic-like pulses as explained in the text.

Our first study was done to compare the intrinsic excitability of the mature and newborn GC models from control (YOUNG) and PILO samples. We compared with the mature GC models only the newborn GC models which have dendrites that reach the outer molecular layer and, consequently, can receive perforant path stimulation (6 YOUNG and 2 PILO GC models). The excitability was assessed via two protocols. The first was used to estimate the minimum depolarizing current pulse of 500 ms duration applied at a proximal dendrite required to evoke a single action potential (rheobase current). The second was used to measure the number of spikes evoked by one depolarizing synaptic-like pulse applied at intervals of 100 ms during 1000 ms at a single distal dendrite (to simulate synapses at the outer molecular layer). The results are shown in Figure 3. The average rheobase current (Figure 3A) was much higher for mature cells than for newborn cells and the average number of spikes evoked by the train of synaptic-like pulses (Figure 3B) was much lower for mature cells than for newborn cells. We also compared the cases with spine loss and without spine loss, representing spine loss by the corrections in membrane resistivity and capacitance mentioned in the methods section. The results of Figure 3 show that the newborn GC models were more excitable than the mature GC models and the effect of spine loss did not change significantly the cell excitability in any of the studied cases.

**Figure 4:**
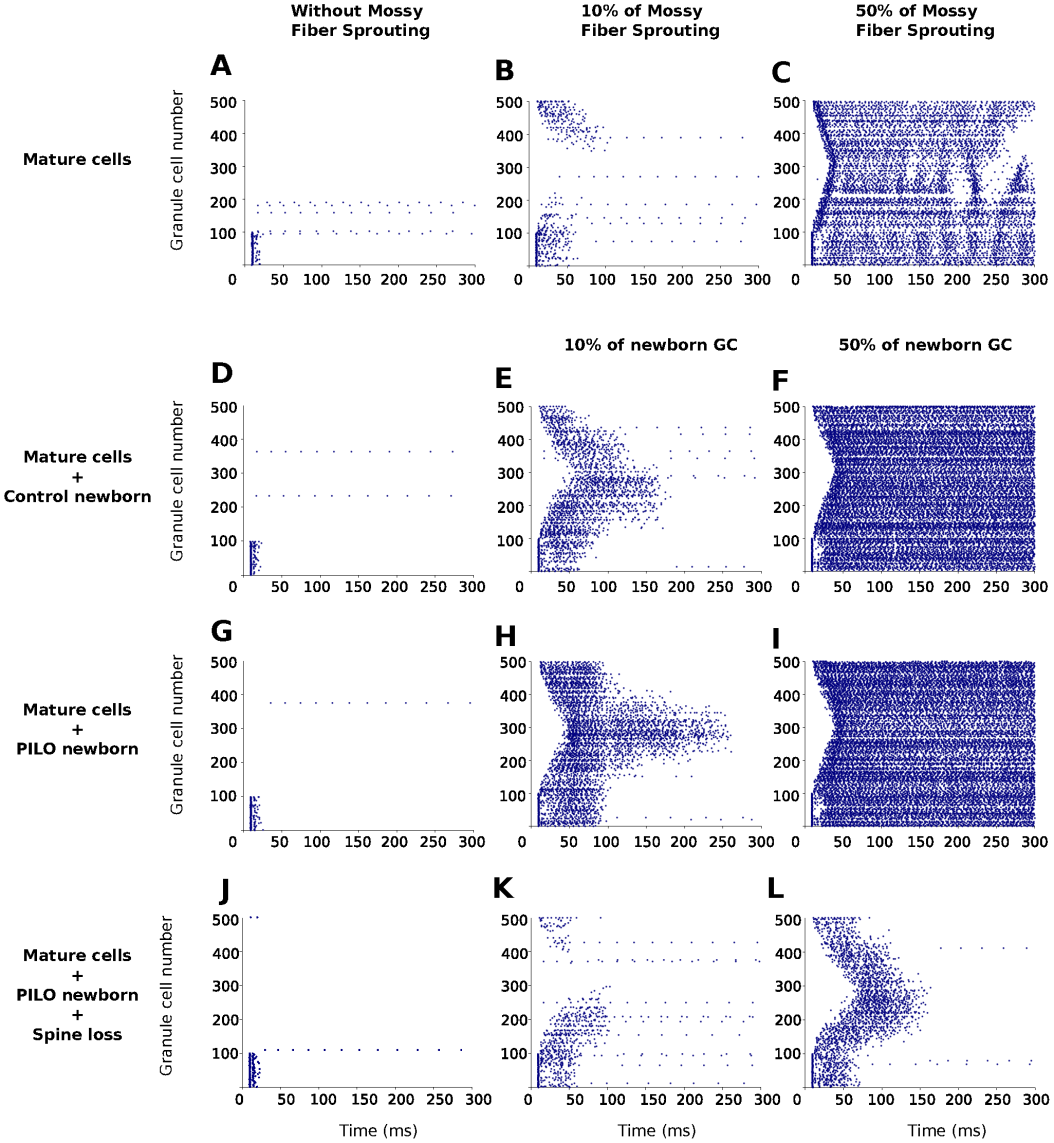
Examples of raster plots for different DG network models. **A**. Response of a mature network without mossy fiber sprouting. **B**. Response of a mature network with 10% mossy fiber sprouting. **C**. Response of a mature network with 50% mossy fiber sprouting. **D**. Response of a mature network with 50% newborn control GCs without mossy fiber sprouting. **E**. Response of a mature network with 10% newborn control GCs and 10% mossy fiber sprouting. **F**. Response of a mature network with 50% newborn control GCs and 10% mossy fiber sprouting. **G**. Response of a mature network with 50% newborn PILO GCs without mossy fiber sprouting. **H**. Response of a mature network with 10% newborn PILO GCs and 10% mossy fiber sprouting. **I**. Response of a mature network with 50% newborn PILO GCs and 10% mossy fiber sprouting. **J**. Response of a mature network with 10% newborn PILO GCs with spine loss and without mossy fiber sprouting. **K**. Response of a mature network with 10% newborn PILO GCs with spine loss and 10% mossy fiber sprouting. **L**. Response of a mature network with 50% newborn PILO GCs with spine loss and 10% mossy fiber sprouting. The latter three figures for the cases with spine loss were obtained for the SL2 case.

The simulations of the DG mature cells networks showed that the insertion of GCs with realistic morphology did not modify the expected response of the DG network model. Our simulations are consistent with the original model of Santhakumatar et al. [28] with and without MFS (Figures 4 A–C). The major difference was in the the speed of propagation of the perforant path stimulation over the DG with realistic models was faster than the one shown by the original DG model. Another difference was the nonuniform activity propagation pattern for 50% of MFS seen in Figure 4C. However, in general terms, the behavior exhibited by the mature DG model matched the expected behavior of the original DG model.

To perform our studies on the effect of the introduction of newborn GCs on the mature DG network, we chose the mature network with 10% of MFS. This case was chosen because this level of sprouting was almost sufficient to produce activity that spread to the entire network (Figure 4B), so we could assess whether the introduction of newborn GCs would cause the network activity to go beyond this underexcited point or not. The results showed that the introduction of newborn cells, both of control (YOUNG) and PILO types, produced an overall increase in DG network activity, but only in combination with MFS (Figures 4 D–I). This effect can be clearly seen by comparing Figures 4D with 4F and 4G with 4I. All raster plots in these four figures were obtained for networks with 50% of newborn GC models, but in the raster plots of Figures 4D and 4G there was no MFS while in the raster plots of Figures 4F and 4I there was 10% of MFS. One can see that in the cases without MFS the activity was restricted to the cells that received perforant path stimulation and died out shortly after that, but in the cases with MFS the activity spread to the whole network and reached a sustained, epileptic-like state. For 10% of MFS, Figures 4E and 4H show that a small amount of newborn GCs was already enough to produce activity that spread to the whole network.

The addition of spine loss to the PILO GC models dramatically reduced the increase of activity produced by the newborn cells in the case with MFS (Figures 4 K–L). In the case with 10% of newborn PILO GCs the activity pattern in the network returned to an underexcited state and in the case with 50% of newborn PILO GCs the activity spread to the entire network but was no longer epileptiform as in the case without spine loss.

**Figure 5:**
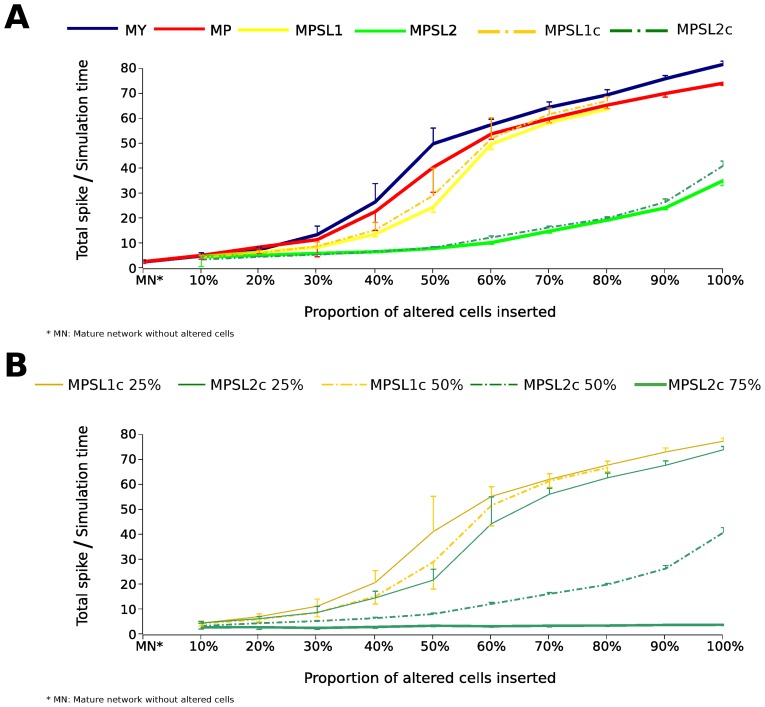
Overall frequency of the network (total spike count over simulation time) for the different network families as a function of the proportion of newborn GCs. All cases are for 10% of mossy fiber sprouting. Each point in the graph corresponds to an average over 20 randomly generated models of the corresponding family. The different network families are indicated by different colors and their codes are the same as defined in Table 2. **A.** Cases in which spine loss was represented by a 50% reduction in the probability of connections with newborn GCs. The MPSL1 network type only had results up to the insertion of 80% of newborn cells because the insertions of 90% and 100% of newborn GCs did not allow the maintenance of the convergence and divergence factors of the network. The error bars represent the standard error. The overall frequency is significantly different over the different proportions (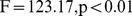) and types of altered cells and spine loss inserted (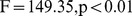) and their interaction (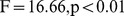). **B.** Cases in which spine loss was represented by a reduction of 25%, 50% and 75% in the probability of newborn GCs receiving connections. The coding is the same as in A but followed by the corresponding reduction in the probability of connection.

To quantify the effect of the introduction of newborn GCs on the activity of the network for the case with 10% of MFS we generated the graphs shown in Figure 5. They give the total number of spikes emitted by the cells of a given network configuration during the simulation time divided by this simulation time, which we will call the overall frequency of the network, as a function of the fraction of newborn GCs introduced in the mature network model. Most of the graphs exhibited the same generic behavior, showing an increase in the overall frequency of the network with the increase in the fraction of newborn cells inserted. Despite the similarities observed among the graphs for the different families of models, the ANOVA test showed significant differences comparing the overall frequency along the different proportions (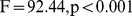) and types (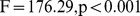) of newborn GCs inserted and their interaction (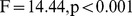).

The curves in Figure 5A for the networks with newborn GCs without spine loss (MY and MP) show that for small fractions (up to 20%) of inserted newborn GCs the overall frequency of the MY network was slightly smaller than the total activity of the MP network (as can be seen in Figures 4E and 4H as well) but for proportions of newborn GCs above 30% the total activity of the MY network became larger than the total activity of the MP network (an effect that is more difficult to see in Figures 4F and 4I). The steeper increases in the overall frequency of the network for these two cases occur for proportions of newborn GCs between 30% and 50% with lower growth rates for proportions of newborn GCs beyond this range.

Incidentally, it is interesting to mention that for proportions of inserted newborn GCs (both YOUNG and PILO) around and above 50% the overall frequency of the network with 10% of MFS became higher than the overall frequency of the mature network with 50% of MFS (data not shown).

Corroborating what was observed in Figures 4K and 4L, Figure 5A shows that the effect of spine loss was to reduce the overall frequency of the network for almost all proportions of inserted newborn GCs. The effect of spine loss was much more sensitive to the maintenance or not of the original convergence and divergence factors of the mature cells in the network than to the way in which spine loss was represented.

In the cases with spine loss in which the convergence and divergence factors of the network were maintained (MPSL1 and MPSL1c), Figure 5A shows that the overall frequency of these network types remained below the overall frequency of the networks without spine loss up to the point in which the proportion of inserted newborn GCs was close to 60%. For proportions of inserted newborn GCs between 60% and 80% the overall frequency of the networks with spine loss became similar to the overall frequency of the network with PILO GCs without spine loss but still below the overall frequency of the network with YOUNG GCs without spine loss. It was not possible to consider proportions of newborn GCs beyond 80% for these cases because it became impossible to maintain the convergence and divergence factors of the mature cells for networks with such large fractions of newborn cells.

The reduction in the overall frequency of the network due to spine loss was much stronger in the cases in which the convergence and divergence factors of network were not maintained (MPSL2 and MPSL2c). In these cases, the overall frequency of the network always remained at or below 50% of the overall frequency of the networks without spine loss. Moreover, in the cases with spine loss and altered divergence and convergence factors of the mature cells the growth of the overall frequency of the network with the proportion of inserted newborn GCs was much slower than in the cases with either no spine loss or spine loss with unaltered divergence and convergence factors of the mature cells. This indicates a strong limiting effect of spine loss over the network excitability in situations in which the rearrangement of connections do not preserve the original convergence and divergence factors of the mature cells that remain in the network.

The effect of altering the probability of connections of newborn PILO GCs with spine loss can be seen in Figure 5B. It shows again the curves of the overall frequency of the network when the probability of connections was reduced by 50% (indicated by MPSL1c 50% and MPSL2c 50%), which are compared to the corresponding curves for a smaller (25%) reduction in the probability of connections and a higher (75%) reduction in the probability of connections. Even when spine loss was not so effective in reducing the number of connections of newborn GCs (the cases with 25% in Figure 5B) the overall frequency of the network remained below the overall frequency of the network for the case without spine loss for most of the proportions of inserted newborn GCs. On the other hand, when spine loss was very effective in reducing the number of connections of newborn GCs (the case with 75% in Figure 5B) the overall frequency of the network remained unchanged at a small value for all proportions of newborn GCs inserted in the network.

## Discussion

The substitution of the simplified GC models by morphologically realistic GC models into the DG model originally proposed by Santhakumar et al. [28] did not cause significant alterations in the DG activity pattern. The networks constituted by mature GC models showed, qualitatively, the same behavior of the original model in both situations with and without MFS. The most important change was in the temporal spike propagation dynamics, which in our adaptation of the model was faster. This change may be due to the way in which we divided the molecular layer, which affected the distribution of recurrent MFS synapses and perforant path synapses from entorhinal cortex. In our model the IML corresponds to the first 17% of the molecular layer measured from the granular cell layer, and is therefore narrower than in the original model of Santhakumar et al. [28]. This means that in our model the MFS synapses, which are placed in the IML, are, on the average, closer to the soma than in the original model, and the perforant path synapses, which are located in the OML, are also, on the average, closer to the soma than in the original model. This makes the GCs to fire faster.

Another change in comparison with the original model of Santhakumar et al. [28] was the nonuniform activity propagation pattern for 50% MFS seen in Figure 4C. This is probably due to the use of GC models with more realistic morphologies in comparison with the simple GC models of Santhakumar et al. [28]. The more complex dendritic trees of our GC models introduce nonlinearities in signal propagation through GC dendrites, which have stronger impact when the number of recurrent GC synapses is high and may be responsible for the observed activity breaks.

The fact that the insertion of newborn GCs without MFS did not provoke an increase in the network activity highlights the importance of MFS in the induction of epileptiform behavior in the network. Otherwise, seizure-induced neurogenesis *per se* is not able to provoke significant changes in the network behavior despite these cells being smaller and therefore more excitable (as shown in Figure 4). Nevertheless, the amount of changes provoked by the simulated neurogenesis may be in the range of the subtle alterations expected from other kinds of processes, such as learning and memory [49], which alter the DG not too much to provoke a seizure but enough to help in the process of codification of new information.

Our simulations showed that the insertion of newborn GCs in a network with MFS provoked a higher increase in the network activity than the one produced by MFS alone. The insertion of the newborn GCs was based on the assumption that these newborn cells preserve the same conditions of the mature cells, with the same number of synapses and the same probability of making connections with other cells. The only difference was in the size of the newborn GC dendrites, which made some cells to not have dendrites extending through the medium or outer molecular layers. In these conditions the interaction between MFS and seizure-induced neurogenesis may produce a more excitable network. Indeed, our results showed that the effect of the newborn GCs can be comparable to the effect of MFS, which is the type of morphological alteration that mostly affects the excitability of the DG circuit, at least in models [28]–[30]. In the previous models, high levels of excitation (epileptic-like) were obtained only for high levels of MFS, viz., the case with 50% of MFS in Figure 4C, but here we showed that similar levels of excitation can be obtained with low levels of MFS (10%) as long as the proportion of newborn GCs in the network is high (>50%). This suggests that newborn GCs have an important role in potentiating the effects of MFS.

However, the insertion of newborn GC models derived from PILO treated animals generated a lower increase of activity compared with the one induced by the insertion of newborn GCs from control animals, in special for cases in which the fraction of inserted newborn GCs was larger than 40% of the total number of GCs. This finding is in agreement with the prediction of the modeling study of Tejada et al. [39], [40], in which it was reported that newborn PILO GCs are less excitable than newborn GCs from control animals. The latter are less excitable than the former when stimulated with a current clamp protocol, and this may be reflected in the slightly smaller increase in the firing rate of the DG network when the newborn GCs inserted on it are of the PILO group in comparison with newborn GCs from the control group. It is possible that the morphological alterations are followed by changes in the ion channels and their maximum conductance distributions, which were not considered in the present study. These ion channels alterations may lead to different firing behaviors than the ones observed in the GCs simulated here with consequences on the excitability of the network but the lack of evidence on the possible changes in the ion channel parameters of newborn GCs from rats that had SE after PILO treatment like the ones considered here leaves this question open.

On the other hand, the inclusion of spine loss in the newborn PILO models provoked a significant reduction in the activity of the circuit for any of the percentages of newborn GCs inserted, even in the case in which the convergence and divergence of the GC models (SL1 models) were maintained. This reduction is consistent with the idea that spine loss acts to maintain homeostasis [19] in the sense that reduction in the number of spines (alone) reduces the number of connections and consequently might decrease the activity of the network. Nevertheless, it is important to point out that our model was specially designed to evaluate the effect of MFS, and in this context, the reduction of spines acts in the opposite direction than the MFS. But it is possible that when other kinds of features are included into the model, such as various types of inhibitory neuronal cells or simulated inputs from medial entorhinal cortex, a pattern in which spine loss does not necessarily reduces the excitability may emerge.

Besides the reduction in the number of connections, spine loss also reduces the membrane surface area of a cell with consequent changes in the membrane resistivity and capacitance [46], [47]. In our simulations, these changes in membrane properties produced small but not significant changes in the excitability, both at the single-cell and the network levels. The factor which had the strongest effect upon the excitability of the dentate network in our simulations was the preservation or not of the network convergence and divergence factors due to spine loss. In network models in which the convergence and divergence factors due to spine loss were not maintained (SL2 models) the excitability was strongly reduced in comparison with the cases in which these factors were maintained (SL1 models). There is no obvious reason for the maintenance of the divergence and convergence factors of the network after the rearrangement of connections following the introduction of newborn GCs with spine loss. Therefore, it is reasonable to assume that the scenario without maintenance of the divergence and convergence factors (SL2 models) is more realistic than the scenario with maintenance of the divergence and convergence factors (SL1 models). We can predict from this that spine loss has a strong protective effect in curtailing the increase of DG activity provoked by the insertion of newborn GCs.

Our computational modeling study has shown that the different neuronal morphological alterations and seizure-induced neurogenesis considered here act much more in combination than as specific features of epileptogenic network activity. On the one hand, there are alterations that increase the number of recurrent connections, such as MFS, having as outcome an increase in the activity of the circuit. On the other hand, alterations such as spine loss can reduce the number of connections and consequently decrease the activity of the network. Along with the above, the DG is constantly exposed to the generation of new cells, which are usually shorter, much more branched [16] and expectedly more excitable, which as consequence potentiate the effect of MFS when inserted into the model. The potentiation provoked by MFS plus neurogenesis may be modulated by seizure-induced dendritic morphological alterations and spine loss, which both reduce the activity of the network. This suggests that the combination of seizure-induced morphological alterations in the apical dendritic tree of newborn GCs and spine loss may have a protective effect on the dentate network against the increase in the activity provoked by MFS and neurogenesis.

Our simulation suggests that the changes in the morphology of GCs provoke diverse effects in the network activity. Some of these changes could be responsible for the increased activity observed in TLE but other could be acting in the opposite direction, decreasing the excitability of the circuit. The balance between these two drives may depend on other factors that were not included in our model, in special, ion channel alterations which may provoke more complex interactions between all of the factors present in an altered circuit.

In relation to the adaptation made in the original model of Santhakumar et al. [28], the changes observed in the activity of the DG model are due solely to the insertion of the different alterations in the GC models, namely altered dendritic tree and spine loss. Therefore, our version respects the topological characteristics of the original model and offers the possibility of studying the interaction of neurogenesis and three of the main morphological alterations usually found in the DG after SE: MFS, changes in the apical dendritic tree of GCs and GC spine loss.

Future studies will obviously add new features to the current modeling, in order to approximate it even more to the complexity and emergent properties of the actual DG and its associated plastic substrates, for example: presence of synapses on basal dendrites [7], [15], [17], DG GC dispersion [3] and ectopic neurogenesis [13].

In conclusion, our findings strongly suggest that the combined presence of morphological features such as MFS, altered apical dendritic tree and spine loss, in a computational model of the DG network, can explain better the inherent complexity of the circuits associated to temporal lobe epileptogenicity. The current network perspective reliably mimics dysfunctional characteristics not necessarily present when simplified or isolated parameters are considered.
